# Meckel’s Diverticulum causing Intussusception in a Newborn

**Published:** 2015-10-01

**Authors:** Dillip Kumar Das, Pravat Kumar Majumdar, Suprabha Shukla

**Affiliations:** Department of Pediatrics, Hi Tech Medical College, Bhubaneswar

**Keywords:** Intussusception, Meckel’s diverticulum, Neonate

## Abstract

A neonate presenting with intussusception probably caused by Meckel’s diverticulum is reported here.

## CASE REPORT

A 20-day-old female neonate was admitted with history of bilious vomiting and refusal to feed for 4 days. There was no history of bleeding per rectum. Baby was born at term, with birth weight 2854 gram and immediate postnatal period was uneventful. On examination, she was dehydrated. Upper abdomen was mildly distended, but no guarding, rigidity, or mass was felt. Digital rectal examination was normal. Differential diagnoses of necrotizing enterocolitis (NEC) and intestinal obstruction were kept in mind. Sepsis work up came negative. On plain x-ray abdomen, multiple air fluid levels were seen with paucity of air in the pelvis and ultrasonography of abdomen and pelvis showed dilatation of bowel loops. After stabilization, exploratory laparotomy was done that revealed jejunoileal intussusception with Meckel’s diverticulum as pathological lead point was found (Fig. 1, 2). The intussusception was manually reduced and end-to-end ileo-ileal anastomosis was done. Postoperative recovery was uneventful.

**Figure:**
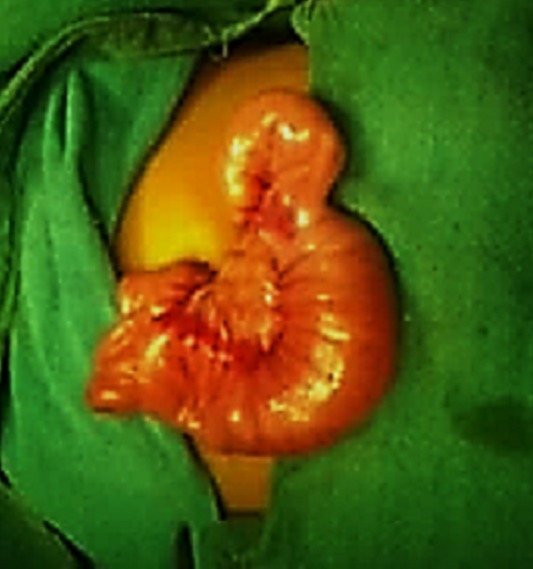
Figure 1: Jejunoileal intussusception.

**Figure:**
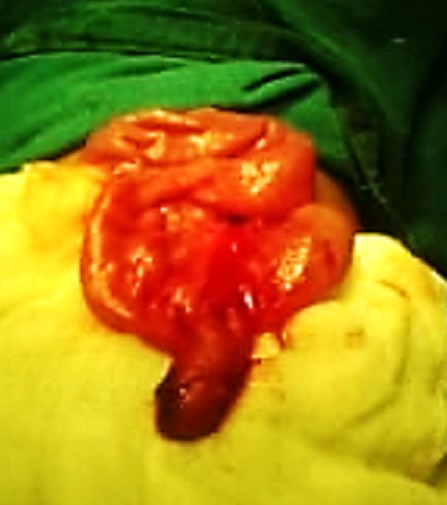
Figure 2: Meckel’s diverticulum delivered after reduction of intussusception.

## DISCUSSION

Meckel’s diverticulum can present at any age group; rarely, a symptomatic Meckel’s diverticulum may present in the neonatal age group [1, 2]. Intussusception occurs very infrequently in the neonatal period, with a reported incidence ranging from 0.3% to 2.7% in the first month of life, and results in less than 3% of all neonatal bowel obstructions [3, 4]. 

Neonatal intussusception does not have any classical radiological signs. The most common imaging findings in neonates with intussusception are signs of ileus such as dilation of bowel loops [4] and occasionally gas fluid levels. Neonatal intussusception having Meckel’s diverticulum is an incidental finding. In our case, the intussusception was jejunoileal and presence of Meckel’s diverticulum may be thought as lead point although it is more common in ileocolic intussusception.

## Footnotes

**Source of Support:** Nil

**Conflict of Interest:** Nil

## References

[1] Sinha CK, Fishman J, Clarke SA. Neonatal Meckel’s diverticulum: spectrum of presentation. Pediatr Emerg Care. 2009; 25:348e9. 10.1097/PEC.0b013e3181a34936 19444035

[2] Slam KD, Teitelbaum DH. Multiple sequential intussusceptions causing bowel obstruction in a preterm neonate. J Pediatr Surg. 2007; 42:1279-81. 10.1016/j.jpedsurg.2007.02.028 17618896

[3] Margenthaler JA, Vogler C, Guerra OM, Limpert JN, Weber TR, Keller MS. Pediatric surgical images: small bowel intussusception in a preterm infant. J Pediatr Surg. 2002; 37:1515-7. 10.1053/jpsu.2002.35437 12378473

[4] Loukas I, Baltogiannis N, Plataras C, Skiathitou AV, Siahanidou S, Geroulanos G. Intussusception in a premature neonate: A rare often misdiagnosed cause of intestinal obstruction. Case Rep Med. 2009;2009:607989. 10.1155/2009/607989 PMC2797753 20049335

